# Evidence for the Immunosuppressive Potential of Calcineurin Inhibitor-Sparing Regimens in Liver Transplant Recipients with Impaired Renal Function

**DOI:** 10.1155/2011/483728

**Published:** 2011-07-06

**Authors:** Kentaro Ide, Yuka Tanaka, Takashi Onoe, Masataka Banshodani, Hirofumi Tazawa, Yuka Igarashi, Nabin Bahadur Basnet, Marlen Doskali, Hirotaka Tashiro, Hideki Ohdan

**Affiliations:** Division of Frontier Medical Science, Department of Surgery, Programs for Biomedical Research, Graduate School of Biomedical Sciences, Hiroshima University, 1-2-3 Kasumi Minami-ku, Hiroshima 734-8551, Japan

## Abstract

Patients requiring liver transplantation (LT) frequently experience renal insufficiency (RI), which affects their survival. Although calcineurin inhibitor-sparing immunosuppressive regimens (CSRs) are well known to prevent RI, the immune state in recipients receiving CSR remains to be intensively investigated. Among 60 cases of living-donor LT at our institute, 68% of the patients had none to mild RI (non-RI group) and 32% of the patients had moderate to severe RI (RI group). The RI group received a CSR comprising reduced dose of tacrolimus, methylprednisolone, and mycophenolate mofetil, while the non-RI group received a regimen comprising conventional dose of tacrolimus and methylprednisolone. One year after LT, the mean estimated glomerular filtration rate (eGFR) in the RI group had significantly improved, although it was still lower than that of the non-RI group. Serial mixed lymphocyte reaction assays revealed that antidonor T-cell responses were adequately suppressed in both groups. Thus, we provide evidence that CSR leads to improvement of eGFR after LT in patients with RI, while maintaining an appropriate immunosuppressive state.

## 1. Introduction

Renal insufficiency (RI) has been widely recognized as a serious complication of liver transplantation that significantly compromises patient outcome [1–4]. Since a number of patients already have varying degrees of RI, including hepatorenal syndrome, before undergoing liver transplantation, and since postoperative standard immunosuppression protocols based on calcineurin inhibitors (CNIs) can lead to severe tubular atrophy, interstitial fibrosis, and focal hyalinosis of the small renal arteries and arterioles, a majority of liver recipients develop some degree of RI [5–7]. An analysis of data from the Scientific Registry of Transplant Recipients indicates that the cumulative incidence of stage 4 [estimated glomerular filtration rate (eGFR) < 30 mL/min/1.73 m^2^] or stage 5 chronic kidney disease (eGFR < 15 mL/min/1.73 m^2^ or need for renal replacement therapy) after liver transplantation is 18% at 5 years [8]. 

Late renal failure is associated with both pre- and posttransplant factors, including higher concentrations of CNIs both early and late posttransplant and can be predicted by creatinine levels in the first year posttransplant [9, 10]. The recognition of these effects induced interest in strategies using a CNI-sparing immunosuppressive regimen (CSR). Current strategies to overcome CNI toxicity include reduction or withdrawal of CNIs concurrent with switching over to less nephrotoxic drugs like the mammalian target of rapamycin (mTOR) inhibitor or mycophenolate mofetil (MMF) [11–17]. Although these strategies have clearly demonstrated the ability to reduce the incidence of nephrotoxicity in various studies, CSR may result in an increased risk for acute rejection episodes in a subset of patients. 

In the present study, we investigated the immune state in liver transplant patients suffering from RI who received a CSR comprising a reduced dose of CNI, methylprednisolone, and MMF. For monitoring the immune-state response to antidonor allostimulation in these patients, we employed a mixed lymphocyte reaction (MLR) assay using an intracellular carboxyfluorescein diacetate succinimidyl ester (CFSE)-labeling technique. By applying the CFSE-based method, the proliferation of viable CD4^+^ and CD8^+^ responder T-cells in response to allostimulation could be separately quantified using multiparameter flow cytometry [18]. The technique allowed us to find that antidonor T-cell responses were adequately suppressed in patients with RI who received the CSR and in patients without RI who received a conventional immunosuppressive regimen.

## 2. Patients and Methods

### 2.1. Patients

Between January 2003 and December 2009, 122 patients underwent living-donor LTs at Hiroshima University Hospital. Of these, 50 patients infected with hepatitis C virus (HCV) and 12 patients who received liver allografts from ABO-blood group incompatible donors were excluded from the study, because they were treated with the diverse immunosuppressive protocols. For the remaining 60 patients, the relationship between RI prior to LT and the clinical/immunological state after LT was investigated. The following information was collected at the time of the transplant: age, sex, etiology of liver disease, model for end-stage liver disease (MELD) score, and diagnosis of hepatocellular carcinoma (HCC) prior to LT. Renal function was evaluated in each participant by determining eGFR. The eGFR of each participant was calculated from their serum creatinine value (SCr) and their age by using the new Japanese equation [19] as follows:

(1) $$\begin{array}{cc} & \text{eGFR}\;\left(\text{mL}/\min\;/1.73\;{\text{m}}^{2}\right)\\ & \;\;=194\times \text{Age}-0.287\\ & \;\;\;\times \text{S}-\text{Cr}-1.094\;\left(\text{if}\;\text{female}\times 0.739\right). \end{array}\;$$

In this study, RI was defined as none to mild (eGFR ≥ 60 mL/min/1.73 m^2^) and moderate (30–59 mL/min/1.73 m^2^) to severe (< 30 mL/min/1.73 m^2^). The MELD score was calculated for each patient using the United Network for Organ Sharing (UNOS) formula based on the laboratory values obtained just prior to LT. Patients were monitored for renal function using serum creatinine level and eGFR at 1, 3, 6, and 12 months after LT.

### 2.2. Immunosuppressive Protocol

**Figure 1:**
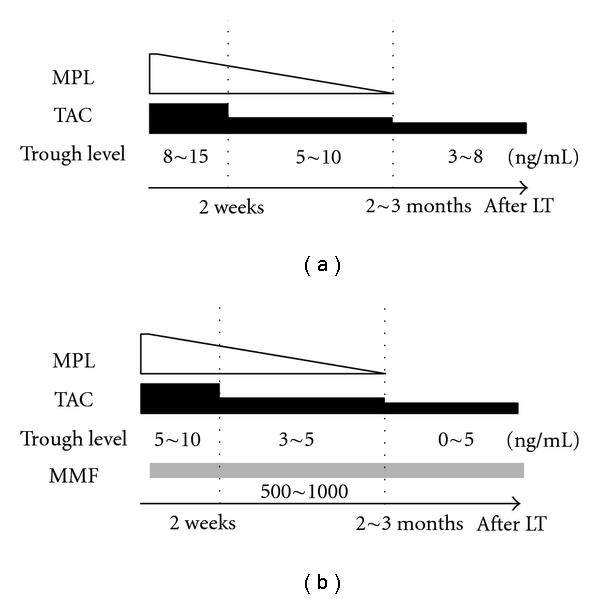
Immunosuppressive protocol after liver transplantation. The basic immunosuppressive regimen comprised tacrolimus (TAC) and methylprednisolone (MPL), with doses gradually being tapered off. The trough whole blood levels of TAC were maintained between 8 and 15 ng/mL in the first few postoperative weeks and between 5 and 10 ng/mL thereafter (a). Renal insufficiency (RI) group received CNI-sparing immunosuppressive regimen (CSR) consisting of TAC reduction and concomitant use of mycophenolat mofetil (MMF) (b).

The basic immunosuppressive regimen after LT for the non-RI group comprised tacrolimus (TAC) and methylprednisolone, with gradual tapering of doses. Patients with RI received a CSR comprising a reduced dose of TAC, methylprednisolone, and MMF (Figure 1). In the conventional regimen, the trough whole blood levels of TAC were maintained between 8 and 15 ng/mL in the first few postoperative weeks and between 5 and 10 ng/mL thereafter. In the CSR, the trough whole blood levels of TAC were maintained between 5 and 10 ng/mL in the first few postoperative weeks and between 3 and 5 ng/mL thereafter.

### 2.3. Immune Monitoring by an In Vitro MLR Assay

For monitoring the immune state, an in vitro MLR assay was performed at 1, 3, 6, and 12 months after LT. Briefly, peripheral blood mononuclear cells prepared from the blood of the recipients, donors, and healthy volunteers with the same blood type as the donors (third-party control) for use as the stimulator cells were irradiated with 30 Gy, and those obtained from the recipients for use as responder cells were labeled with 5 lm CFSE (Molecular Probes Inc., Eugene, OR, USA), as described previously [18]. The stimulator and responder cells were incubated for 5 days. CFSE stably stains intracellular proteins without causing toxicity, and the fluorescence intensity of each stained cell segregates equally among daughter cells during cell division, resulting in sequential halving of the cellular fluorescence intensity with every successive generation. After culturing for MLR, the harvested cells were stained with either phycoerythrin- (PE-) conjugated antihuman CD4 or PE-conjugated antihuman CD8 monoclonal antibodies and subjected to analysis by flow cytometry. All analyses were performed on a FACSCalibur flow cytometer (Becton Dickinson, Mountain View, CA, USA). T-cell proliferation was visualized by the serial-halving of the fluorescence intensity of CFSE. CD4^+^ and CD8^+^ T-cell proliferation and stimulation index were quantified using a method described previously [18].

### 2.4. Statistical Analysis

Quantitative variables were expressed as mean ± standard deviation (SD) or median (range). Categorical variables were presented as values and percentages. Student's *t*-test, Mann-Whitney test, chi-square test, and Fischer's exact test were used to compare variables between the two groups. Paired *t*-tests were performed to compare continuous variables throughout the study period. The Kaplan-Meier analyses were used to compare time-to-event variables. *P* Values < 0.05 were considered statistically significant.

## 3. Results

The 60 patients included 34 males and 26 females; their ages ranged from 20 to 69 (median 52) years. The primary diseases in these patients included hepatitis B virus-related cirrhosis in 24 patients (of these, 18 patients had HCC), alcoholic cirrhosis in 13 patients (of these, 6 patients had HCC), autoimmune hepatitis in 5 patients (of these, 1 patient had HCC), and other diseases in 18 patients. 

**Figure 2:**
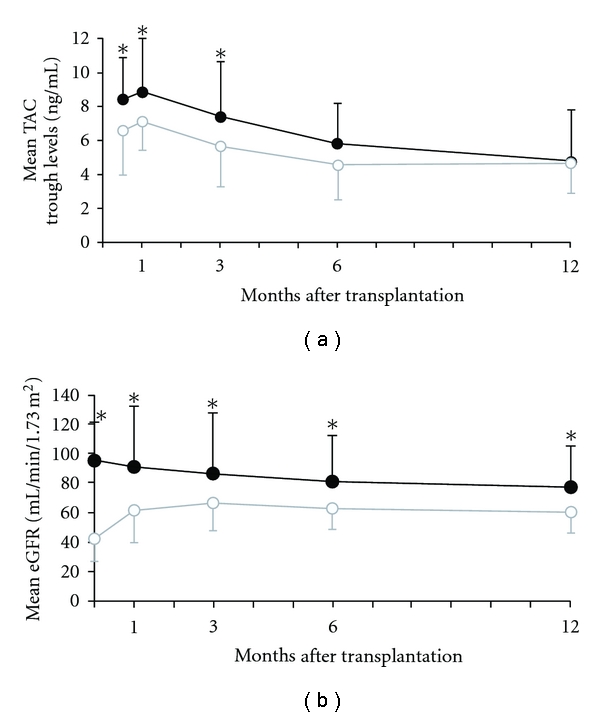
Kinetics of mean trough levels of tacrolimus and mean estimated glomerular filtration rate (eGFR) in the RI group and non-RI group during the first year after transplantation. (a) Mean trough levels of tacrolimus in the non-RI group (black line) and RI group (gray line). (b) Mean estimated glomerular filtration rate (eGFR) in the non-RI group (black line) and RI group (gray line). Data are median ± SD of values. *P < 0.05.

Before the LTs, 68% of the patients had none to mild RI (non-RI group; mean eGFR, 94.8 ± 26.9 mL/min/1.73 m^2^) and 32% of the patients had moderate to severe RI (RI group; mean eGFR, 42.5 ± 15.9 mL/min/1.73 m^2^). The characteristics of these patients are listed in Table 1. There was a difference in MELD score between the groups. Mean TAC trough levels during the first year after LT in the non-RI and RI groups are shown in Figure 2(a). There were differences in mean TAC trough levels during 3 months after LT between the groups. One year after the LDLTs, the mean eGFR in the non-RI group had significantly deteriorated (from 94.8 ± 26.9 to 77.2 ± 28.2 mL/min/1.73 m^2^, *P* < 0.01). In contrast, the mean eGFR in the RI group had significantly improved after LT (from 42.5 ± 15.9 to 60.1 ± 13.5 mL/min/1.73 m^2^, *P* < 0.01), although it was still lower than that of the non-RI group (Figure 2(b)). Notably, 53% of the patients in the RI group were completely cured of RI by 1 year after LT. None of the patients had severe RI at 1 year after LT nor required chronic hemodialysis during the observation period. 

**Figure 3:**
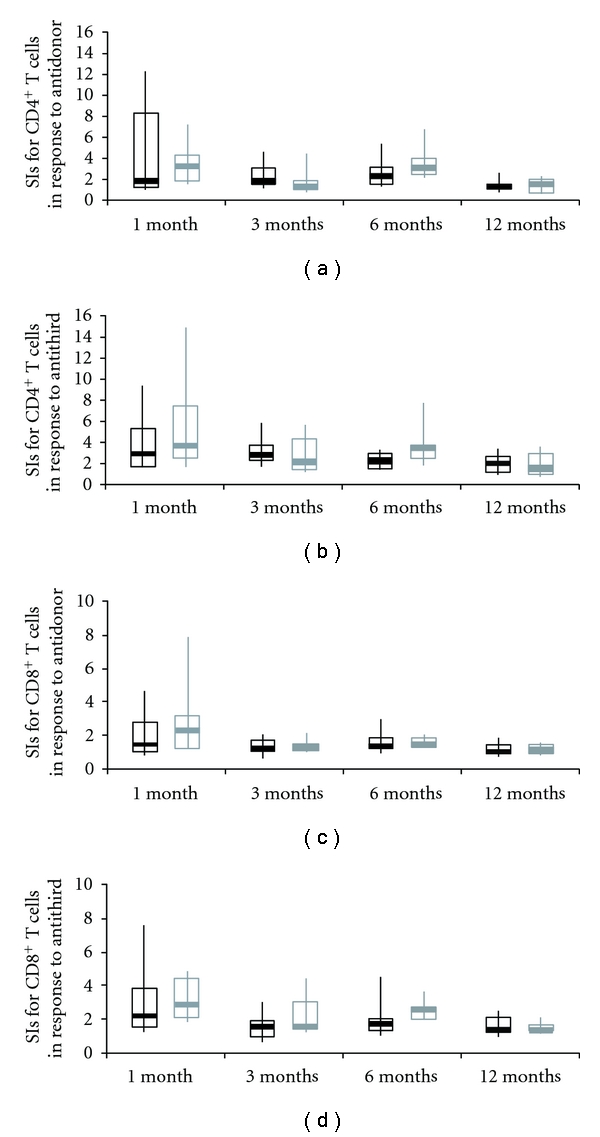
Kinetics of stimulation index in the RI group and non-RI group during the first year after transplantation. Stimulation index (SI) of each of the CD4^+^ T-cell (a, b) and CD8^+^ T-cell (c, d) subsets in the antidonor (a, c) and anti-third-party (b, d) MLR in patients in non-RI group (black line) and RI group (gray line). CD4^+^ and CD8^+^ T-cell proliferation and their SIs were quantified as follows. The number of division precursors was extrapolated from the number of daughter cells of each division, and the number of mitotic events in each of the CD4^+^ and CD8^+^ T-cell subsets was calculated. Using these values, the mitotic index was calculated by dividing the total number of mitotic events by the total number of precursors. The SIs of allogeneic combinations were calculated by dividing the mitotic index of a particular allogeneic combination by that of the self-control. The box plot represents the 25th to 75th percentile, the dark line is the median, and the extended bars represent the 10th to the 90th percentile.

To evaluate the immune status of these patients, we employed a serial MLR assay using a CFSE-labeling technique. Lack of proliferation of both CD4^+^ and CD8^+^ T-cells in the antidonor CFSE-MLR assay indicates suppression of the antidonor response, whereas a remarkable proliferation of these T-cells reflects a strong antidonor response. In both groups, limited CD4^+^ and CD8^+^ T-cell proliferation was observed in the antidonor responses as compared with the anti-third-party responses through the first year. At 1 month after LT, the average of stimulation index (SI) for CD4^+^ T-cells in response to anti-third-party stimulation was > 2 (the average value in healthy volunteers without any immunosuppressive treatment) that is, there was a normal response in the anti-third-party (Figures 3(a) and 3(b)). At 1 year after LT, the average of SIs for CD4^+^ and CD8^+^ T-cells in response to both antidonor and anti-third-party stimulation was < 2 (Figures 3(c) and 3(d)). There were no significant differences in acute rejection rates, bacterial, fungal, or cytomegalovirus infection rates and patient survival between the groups (Table 1).

## 4. Discussion

Chronic RI is a serious complication in liver transplantation that significantly compromises patient survival and outcome. Depending on the criteria applied for a definition of chronic renal insufficiency and the duration of followup, the reported rate of chronic renal insufficiency after liver transplantation may vary from 10% to 80% [1, 20–22]. CNI toxicity has been defined as one of the possible risk factors for renal insufficiency in long-term liver transplant survivors. It has been shown that exposure to CNIs within the first 6 months after liver transplantation represents a risk factor for renal failure [23]. The GFR at 1 year had a better correlation with later renal function than the pretransplant GFR [24]. The recognition of these facts induced interest in preventing CNI toxicity. It has also reported that the use of adjunctive MMF immediately after LT might protect against CNI nephrotoxicity, potentially without the need for dose reduction or increased risk of adverse events [25]. Therefore, current strategies to overcome CNI toxicity include reduction or withdrawal of CNIs along with switching to mTOR inhibitor or MMF-based regimens [11, 12, 14, 15, 26–28]. These strategies have been documented in several recent and ongoing trials to achieve an improvement in renal function in a large proportion of liver transplant patients. 

In our CSR using MMF, wherein our study results agree with the results from previous studies, patients with pre-transplant renal insufficiency were associated with less impairment of renal function without an increased frequency of rejection, infection, or patient survival. In addition to this clinical evidence for the usefulness of the CSR using MMF, the present study provides immunological evidence, by analyzing the data obtained from an MLR assay, that antidonor T-cell responses were adequately suppressed in patients who received the CSR and in patients who received the conventional immunosuppressive regimen. Notably, the individual variations of SIs of CD4^+^ T-cell and CD8^+^ T-cell subsets on antidonor T-cell responses in patients who received the CSR were smaller than those in patients who received the conventional regimen, although the average values of both were similar. This might be explained by the possibility that the CSR comprising triple immunosuppressive drugs was equally effective in a wide variety of patients. 

Several limitations of this study are present. Our sample size was relatively small without long-term followup, and single-center retrospective data are reported. Since the 2 groups of patients are not perfectly comparable as renal impairment can reduce immune responses, we could not rule out a possibility that reduced CNI, without necessarily adding MMF, may be sufficient for the treatment of these patients. 

We excluded HCV positive cases and ABO-blood group incompatible cases from the study because of diverse protocol (In brief, in patients with HCV infection, methylprednisolone is not administered, which may be beneficial for preventing enhanced viral replication. Instead, basiliximab and MMF are usually administered to such patients. In ABO-blood group incompatible cases, anti-CD20 monoclonal antibody is administered for eliminating temporarily B cells 2 weeks before transplantation, and simultaneously commencing administration of CNI and MMF.). Hence, the effect of CSR in RI patients with those backgrounds remains to be elucidated. Nevertheless, this first evaluation of the immune state in liver transplant patients suffering from RI received a CSR was essential before to propose an evaluation at a larger scale. 

In conclusion, patients with pre-transplant RI receiving CSR under immunological monitoring using an MLR assay were associated with less impairment of renal function without an increased frequency of rejection or patient survival. Antidonor T-cell responses were adequately suppressed in these patients as well as in patients who received the conventional immunosuppressive regimen comprising a standard dose of CNI.

## Figures and Tables

**Table 1:** Patient characteristics at living donor liver transplantation.

	Non-RI group (*n* = 41)	RI group (*n* = 19)	*P* value
(eGFR (mL/min/1.73 m^2^))	(94.8 ± 26.9)	(42.5 ± 15.9)	
Age at LT (years)	49.2 ± 11.5	52.9 ± 9.0	0.23
Male sex—*n *(%)	21 (51.2)	13 (68.4)	0.21
Primary diagnosis—*n* (%)			0.63
HBV	15 (36.6)	9 (47.4)	
Alcoholic	8 (19.5)	5 (26.3)	
AIH	4 (9.8)	1 (5.3)	
Others	14 (34.1)	4 (21.1)	
MELD	16.5 ± 7.1	24.7 ± 10.7	< 0.01
eGFR at 1st year after LT (mL/min/1.73 m^2^)	77.2 ± 28.2	60.1 ± 13.5	< 0.01
eGFR > 60 at 1st year after LT—*n* (%)	26 (72.2)	10 (58.8)	0.33
AR within 1st year—*n* (%)	10 (24.4)	5 (26.3)	0.87
Bacterial infections—*n* (%)	13 (31.7)	8 (42.1)	0.43
Fungal infections—*n* (%)	4 (9.8)	4 (21.1)	0.23
CMV infections—*n* (%)	10 (24.4)	7 (36.8)	0.32

RI, renal insufficiency; LT, liver transplantation; HBV, hepatitis B virus; AIH, Autoimmune hepatitis; eGFR, estimated glomerular filtration rate; MELD, model for end-stage liver disease; AR, acute rejection; CMV, cytomegalovirus. Data are expressed as means ± standard deviation. Difference with *P* < 0.05 was considered significant.

## Abbreviations

AIH: Autoimmune hepatitis
AR: acute rejection
CFSE: carboxyfluorescein diacetate succinimidyl ester
CMV: cytomegalovirus
CNI: calcineurin inhibitor
CSR: CNI sparing immunosuppressive regimen
eGFR: estimated glomerular filtration rate
HBV: hepatitis B virus
HCV: hepatitis C virus
LT: liver transplantation
MELD: model for end-stage liver disease
MLR: mixed lymphocyte reaction
MMF: mycophenolate mofetil
mTOR: mammalian target of rapamycin
MPL: methylprednisolone
RI: renal insufficiency
SI: stimulation index
TAC: tacrolimus.
